# A Multifunctional Therapeutic Platform: Ce/Zn/Sr-Doped Mesoporous Bioactive Glass Nanoparticles for Bone Repair

**DOI:** 10.3390/ijms27062640

**Published:** 2026-03-13

**Authors:** Nattakan Sae-Sue, Wen-Ta Su, Poommaree Namchaiw, Kamolchanok Ngamkham, Nattida Suwanakitti, Parichart Naruphontjirakul

**Affiliations:** 1 Biological Engineering Program, Faculty of Engineering, King Mongkut’s University of Technology Thonburi, Bangkok 10140, Thailand; nattaporn.sae-@mail.kmutt.ac.th (N.S.-S.); poommaree.nam@mail.kmutt.ac.th (P.N.); kamolchanok.nga@mail.kmutt.ac.th (K.N.); 2 Department of Chemical Engineering and Biotechnology, National Taipei University of Technology, Taipei 106344, Taiwan; f10549@ntut.edu.tw; 3 Biorefinery and Bioproduct Technology Research Group, National Center for Genetic Engineering and Biotechnology, Pathum Thani 12120, Thailand; nattida.suw@biotec.or.th

**Keywords:** mesoporous bioactive glass nanoparticles, nanoceria, antibacterial, antioxidant, bone regeneration

## Abstract

Mesoporous bioactive glass nanoparticles (MBGNs) are promising for bone tissue engineering; however, surgical site infection and oxidative stress often compromise regeneration. To address this, MBGNs co-doped with cerium (Ce), zinc (Zn), and strontium (Sr) were synthesized using a microemulsion-assisted sol-gel route (xCe-yZn-Sr-MBGNs; x = 0, 1, 2; y = 0, 0.5, 1). The resulting spherical nanoparticles (150–200 nm) exhibited a mesoporous structure with a specific surface area of (~340–425 m^2^/g), sustained ion release, and apatite formation in simulated body fluid. In vitro evaluations with MC3T3-E1 pre-osteoblasts demonstrated dose-dependent cytocompatibility, specifically in the co-doped formulations; however, higher Ce concentrations (2Ce-yZn-Sr-MBGNs) reduced viability following prolonged exposure. Crucially, the 1Ce-1Zn-Sr-MBGNs significantly enhanced osteogenic differentiation, as evidenced by a two-fold increase in osteogenic marker gene expression and a ~45% increase in calcium mineral deposition compared to undoped MBGNs within 14 days. Moreover, these particles accelerated cell migration, achieving ~70% scratch-wound closure within 24 h. Furthermore, 1Ce-1Zn-Sr-MBGNs displayed strong radical scavenging capacity and potent antibacterial activity against *S. aureus* and *P. aeruginosa*. These findings indicated that 1Ce-1Zn-Sr-MBGNs exhibited multiple therapeutic effects, including antibacterial, radical-scavenging, and osteogenic effects. By optimizing dopant ratios, these multifunctional nanomaterials emerge as promising candidates for next-generation bone grafts or implant coatings. Within the scope of this study, they demonstrated the capacity to simultaneously address three critical challenges in bone healing: controlling infection, mitigating oxidative stress, and promoting mineralized tissue formation. While these in vitro results provide a robust foundation, further in vivo validation is warranted to confirm their efficacy within complex physiological environments.

## 1. Introduction

Despite the prevalence of bone fractures, exceeding 450 million cases globally [1], traditional grafting and surgical interventions are often limited by donor-site morbidity and infection risks [2]. Critically, the innate regenerative capacity of bone is significantly impaired by oxidative stress. The accumulation of reactive oxygen species (ROS) damages bone cells and delays the repair process [3,4].

Addressing these challenges requires innovative biomaterials strategies to enhance bone repair and regeneration. Key attributes of biomaterials include biocompatibility, which reduces toxicity and adverse immune responses, biodegradability, which minimizes long-term complications, and bifunctionality, which replicates biological properties [5,6]. Mesoporous bioactive glass nanoparticles (MBGNs) have emerged as a superior platform compared to traditional micro-sized or dense bioactive glasses due to their high surface area and tunable interconnected mesoporous network [7]. These intrinsic characteristics allow for efficient bioactivity and the precise incorporation and release of therapeutic agents, making them ideal for multifunctional applications in tissue engineering [8,9].

The biological performance of MBGNs is intrinsically linked to their chemical composition and glass network structure. Binary SiO_2_-CaO systems are particularly favored for their simplicity and high bioactivity compared to pure silica. Calcium acts as a network modifier that disrupts the siloxane (Si-O-Si) framework, creating non-bridging oxygens (NBOs). While the 70% SiO_2_-30% CaO (molar ratio) system is a standard benchmark for stability and bioactivity, increasing the ratio of network modifiers can further enhance reactivity. A shift towards a lower silica and higher network modifier content typically decreases the network connectivity. Theoretically, this reduction in connectivity should facilitate faster glass degradation and more rapid release of therapeutic ions, which are critical signaling molecules for osteoblast proliferation and differentiation [9,10].

Incorporating therapeutic ions such as magnesium (Mg), strontium (Sr), zinc (Zn), cerium (Ce), or silver (Ag) into the silica-calcium network of SiO_2_-CaO systems allows for the precise tailoring of biological responses. These multifunctional MBGNs can effectively modulate cellular behavior, promote osteogenic differentiation, and provide potent antibacterial effects, thereby reducing the risk of post-surgical infections [8,9,10,11,12]. Sr-doped MBGNs have been reported to stimulate osteoblast activity and upregulate key osteogenic markers, including collagen Type I (*COL1*) and osteocalcin (*OCN*). Furthermore, the substitution of Ca with Sr can modulate the zeta potential of the nanoparticles, thereby enhancing the adsorption of negatively charged proteins onto the bioactive surface [8,13]. Zn-doped MBGNs promoted osteogenic differentiation and accelerated the proliferation of fibroblasts and epithelial cells, while simultaneously providing potent antibacterial activity and modulating immune responses by inhibiting pro-inflammatory macrophage polarization [14,15]. Ce-doped MBGNs represented a unique class of therapeutic materials capable of mimicking enzyme-like antioxidant activity. At physiological pH, Ce scavenges ROS through its ability to undergo reversible transitions between Ce^3+^ and Ce^4+^ oxidation states. This redox behavior induces anti-inflammatory responses and supports osteoblastic lineage progression; conversely, in acidic microenvironments characteristic of bacterial infection, Ce acts as a pro-oxidant, exerting robust antimicrobial effects [16,17].

While the existing literature has extensively detailed the use of various dopants to improve the bioactivity of mesoporous bioactive glass nanoparticles (MBGNs), the multifunctional potential of a triple-doped Ce-Zn-Sr system remains unexplored. A major challenge in this area arises from dose-dependent toxicity and narrow therapeutic windows of dopant ions, as inappropriate concentrations can induce cytotoxicity. Thus, achieving an optimal balance between therapeutic efficacy and potential toxicity is critical. Optimizing the composition, particle size, and ion-doping strategy to attain targeted biological responses remains a key research focus. This study addresses the dual challenge of maximizing therapeutic efficacy, specifically antibacterial, antioxidant, and osteogenic activities, while navigating the narrow safety margins of ion-doping. Through a microemulsion-assisted sol-gel approach, xCe-yZn-Sr-MBGNs were synthesized to optimal compositional windows for targeted biological performance. These findings elucidate the relationship between co-dopant concentration and cellular response, providing a rational design framework for multifunctional, highly biocompatible MBGNs for advanced bone regeneration applications.

## 2. Results and Discussion

The xCe-yZn-Sr-MBGNs were successfully synthesized using a microemulsion-assisted sol-gel method, which comprises a surfactant, an oil phase, and an aqueous phase. In this study, cetyltrimethylammonium bromide (CTAB) was employed as the surfactant to generate a mesoporous structure, while ethyl acetate was used as the oil phase to enhance dispersity and prevent aggregation by acting as a steric barrier. Nanoparticle formation occurred in the aqueous phase through simultaneous hydrolysis and polycondensation of the silica precursor tetraethyl orthosilicate (TEOS) in the presence of cerium precursors and a base catalyst (ammonia solution), which is critical for initiating nanoparticle nucleation [18]. Subsequent incorporation of cations, including Ca, Sr, and Zn, was achieved via a post-modification approach, followed by calcination to facilitate their incorporation into the silica network. The resulting nanoparticles were washed to remove residual salts and subjected to crystallization from the Zn precursor prior to further characterization. The composition of the xCe-yZn-Sr-MBGNs was systematically varied (x = 0, 1, 2 mol% for Ce and y = 0, 0.5, 1 mol% for Zn) based on established safety thresholds in the literature. Specifically, Ce concentrations were maintained below 5 mol% to avoid interference with mitochondrial activity [19,20], while Zn levels were kept below 2 mol% to optimize osteoblast proliferation and avoid the induction of apoptosis [21,22]. At these controlled levels, the interactive release of ions could remain within a biocompatible range, effectively addressing the safety concerns often associated with the narrow therapeutic windows of metallic dopants. By operating within these conservative ranges, the xCe-yZn-Sr-MBGNs system balances therapeutic efficacy with a high margin of translational safety.

### 2.1. xCe-yZn-Sr-MBGNs Physicochemical Properties

**Figure 1:**
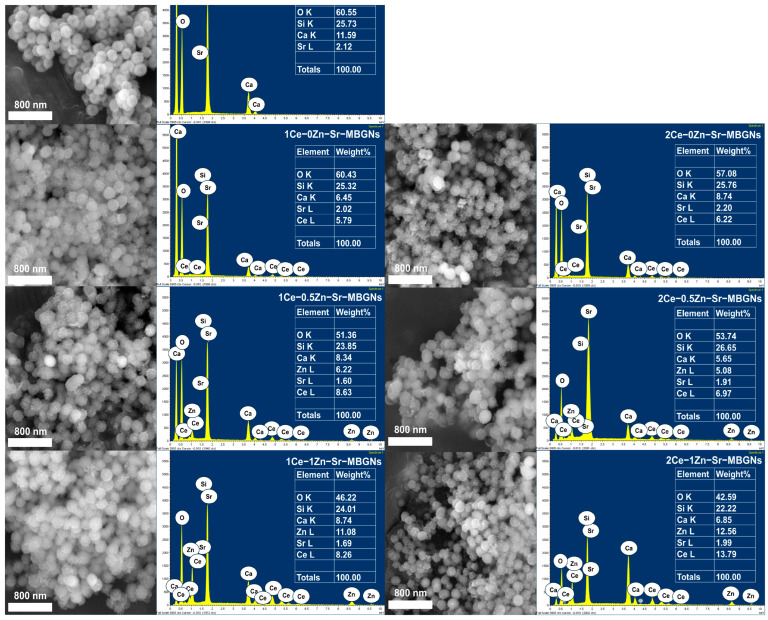
SEM images and EDS analysis of xCe-yZn-Sr-MBGNs with scale bar = 800 nm.

The size and morphological characteristics of the nanoparticles were evaluated using DLS and SEM (Table 1 and Figure 1). As summarized in Table 1, the particle size measured by DLS was consistently larger than that observed by SEM; this is in agreement with previous reports [23], which demonstrated that DLS measurements of MBGNs tend to exceed SEM measurements. DLS assesses the random motion of particles in liquid suspension to determine their hydrodynamic diameter, which encompasses the nanoparticle core, adsorbed ions, and the hydration shell, thereby yielding larger values compared with SEM [24]. SEM analysis (Figure 1) revealed that the xCe-yZn-Sr-MBGNs were uniform, monodisperse, and spherical particles with sizes ranging from 150 to 200 nm. The EDS elemental analysis showed that Sr, Zn, and Ce were successfully incorporated into the xCe-yZn-Sr-MBGNs. The incorporation of cerium at low levels (1Ce-yZn-Sr-MBGNs) did not significantly alter particle size; however, increasing the cerium content to 2Ce-yZn-Sr-MBGNs resulted in a slight reduction in particle size to approximately 170 nm. This slight reduction in particle size may be attributed to the addition of the Ce precursor during the sol-gel process, which can influence the nucleation mechanism. Cerium interacts with silanol groups, thereby modifying the hydrolysis and condensation reactions and resulting in a slower particle formation rate. Additionally, the Ce precursor acts as a nucleation promoter by modifying the silica network structure and inducing localized charge imbalances. These disrupted regions create energetically favorable sites for nucleation, thereby enhancing the nucleation rate. However, an increased nucleation rate limits the amount of precursor material (TEOS) available for the growth of each particle, resulting in smaller particle sizes [25,26]. This observation was consistent with a previous study, which reported that the size of Ce-MBGNs did not change significantly after the addition of 1 mol% Ce precursor during the sol-gel process [16]. Similarly, in the present study, the sizes of 0Ce-0Zn-Sr-MBGNs and 1Ce-0Zn-Sr-MBGNs were not significantly different. A low concentration of Ce precursor may disrupt hydrolysis and condensation processes, but its effect on nucleation appears minimal, yielding no substantial change in particle size compared to 2Ce-yZn-Sr-MBGNs. In contrast, nanoparticle size decreases with increasing Ce content in the form of nanoceria [20]. This finding aligns with the slight size reduction observed in 2Ce-yZn-Sr-MBGNs upon increasing the Ce precursor content.

Zeta potential measurements were performed to assess nanoparticle stability at pH 6.5 (Table 1). Zeta potential reflects the surface charge at the shear slipping plane, with values >+30 mV or <−30 mV generally preventing particle agglomeration through electrostatic repulsion [27]. The undoped 0Ce-0Zn-Sr-MBGNs exhibited a zeta potential of −27.5 ± 1.4 mV near the typical stability threshold. Doping with cerium (Ce) decreased the negative surface charge to −21.3 ± 1.0 mV (1Ce-0Zn-Sr-MBGNs) and −18.8 ± 0.8 mV (2Ce-0Zn-Sr-MBGNs). Further incorporation of Zn shifted the zeta potential toward less negative values, reaching −14.1 ± 1.5 mV for 1Ce-0.5Zn-Sr-MBGNs and −13.1 ± 0.7 mV for 1Ce-1Zn-Sr-MBGNs, indicating partial neutralization of silanol (Si-OH) groups by cations. These trends are consistent with previous reports demonstrating that cation doping increases zeta potential through network modification [12,20]. Surface charge is a key determinant of cellular uptake and nanocarrier performance. In this study, all particles retained a negative zeta potential, which favors osteoblast adhesion and proliferation. Negatively charged surfaces facilitate adsorption of positively charged proteins, enhancing cellular adhesion and chemotaxis [28]. Consistently, previous studies reported that negatively charged MBGNs exhibit greater internalization by MC3T3-E1 pre-osteoblasts compared with neutral or positively charged counterparts after 24 h [23].

**Figure 2:**
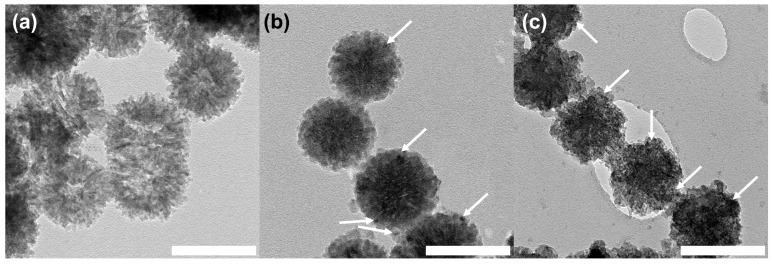
TEM image of (**a**) 0Ce-0Zn-Sr-MBGNs, (**b**) 1Ce-0Zn-Sr-MBGNs, and (**c**) 2Ce-0Zn-Sr-MBGNs, highlighting the presence of nanoceria within the particle matrix (White arrow). Scale bar = 200 nm.

The surface and porous structure of nanoceria was observed in 1Ce-0Zn-Sr-MBGNs and 2Ce-0Zn-Sr-MBGNs (Figure 2), whereas the Ce-free particles (0Ce-0Zn-Sr-MBGNs) lacked these features, indicating that nanoceria formation occurred during the sol-gel process upon addition of the Ce precursor. This aligns with previous reports showing that one-pot, ultrasound-assisted sol-gel synthesis yields ultrasmall nanoceria within the silica network [20]. Furthermore, post-functionalization cation doping did not significantly alter particle size or morphology, likely because the silica network was already established. In this method, nanoparticles were soaked in Ca, Sr, and Zn precursors to promote surface adsorption, followed by calcination to incorporate the ions into the silica framework. As a result, cation incorporation via post-functionalization preserves the morphological properties of nanoparticles formed through hydrolysis and polycondensation during the sol-gel process [18].

**Figure 3:**
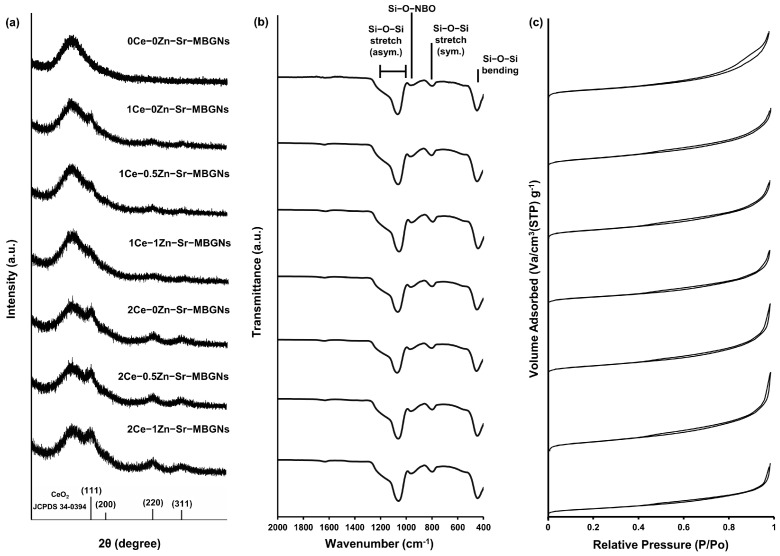
(**a**) XRD, (**b**) FTIR spectra, and (**c**) N2 adsorption−desorption isotherm of xCe-yZn-Sr-MBGNs.

X-ray diffraction (XRD) analysis was performed to investigate the crystallographic structure of the xCe-yZn-Sr-MBGNs (Figure 3a). All xCe-yZn-Sr-MBGN samples exhibited a broad halo between 20 and 30° (2θ), confirming the amorphous nature of the silicate network. This amorphous structure enhanced degradability, facilitating ion release from the silica matrix, which promoted cellular activity and bioactivity through ion exchange [29,30]. Ce-doped samples (1Ce-yZn-Sr-MBGNs and 2Ce-yZn-Sr-MBGNs) displayed additional peaks at 28.5° (111), 47° (220), and 56° (311) (JCPDS no. 34-0394), while retaining the amorphous silicate pattern, indicating the presence of nanoceria within the particles, consistent with TEM observations (Figure 2) [20,31]. The peak intensity of 2Ce-yZn-Sr-MBGNs was higher than that of 1Ce-yZn-Sr-MBGNs, suggesting that higher Ce precursor concentrations enhance nanoceria formation. The presence of crystalline CeO_2_ phases was confirmed by XRD analysis. When combined with EDS results and the nanoscale particle size observed by TEM, these findings demonstrate the successful formation and incorporation of CeO_2_ within the xCe-yZn-Sr-MBGNs. Nanoceria is known to act as a radical scavenger by mitigating oxidative stress and potentially improving osteogenesis and the therapeutic performance of MBGNs [19]. Based on these findings, xCe-yZn-Sr-MBGNs containing nanoceria were hypothesized to exhibit enhanced antioxidant activity. In contrast, Zn doping did not alter the XRD patterns. The diffraction profiles of 1Ce-0.5Zn-Sr-MBGNs and 1Ce-1Zn-Sr-MBGNs were similar to 1Ce-0Zn-Sr-MBGNs, and similarly, 2Ce-0.5Zn-Sr-MBGNs and 2Ce-1Zn-Sr-MBGNs matched 2Ce-0Zn-Sr-MBGNs. These results indicated that Zn acts as a network modifier incorporated through ionic bonding rather than forming bridging oxygen bonds, and thus, varying Zn content does not affect the crystalline structure of the particles.

Fourier-transform infrared (FTIR) spectroscopy was used to investigate the molecular structure and chemical bonding of the xCe-yZn-Sr-MBGNs (Figure 3b). All samples exhibited similar spectral patterns characteristic of a silica network, with peaks at 1000–1200 cm^−1^ and ~800 cm^−1^ corresponding to asymmetric and symmetric stretching vibrations of Si-O-Si bonds, and a band at 450–460 cm^−1^ assigned to Si-O-Si bending vibrations. These features were consistent with typical bioactive glass spectra and corroborate the amorphous silicate structure observed in XRD analysis [32]. A weak peak at 930–970 cm^−1^, attributed to Si-O non-bridging oxygen (NBO), indicated successful incorporation of cations such as Ca, Sr and Zn acting as network modifiers [12]. Notably, while EDS elemental analysis confirmed that Sr, Zn, and Ce were successfully incorporated into the xCe-yZn-Sr-MBGNs (Figure 1), the intensity and position of the NBO-associated FTIR band remained relatively stable across samples. This suggests that the primary silicate backbone remains dominant, and the subtle structural changes induced by increasing dopant concentrations may be partially masked by the overlapping, high-intensity asymmetric stretching vibrations of the Si-O-Si network. Incorporation of Ce induced a blue shift in the asymmetric Si-O-Si stretching from 1067 cm^−1^ (0Ce-0Zn-Sr-MBGNs), reflecting changes in the chemical environment, bonding interactions, or vibrational frequency, without disrupting the silica network [33]. This shift likely arises from the formation of nanoceria and integration of other cations, confirming their successful incorporation while preserving the amorphous structure.

Overall, morphological and physicochemical analyses demonstrated that xCe-yZn-Sr-MBGNs with ~200 nm diameter were successfully synthesized via the microemulsion-assisted sol-gel and post-functionalization methods. The Ce precursor facilitated nanoceria formation within the silica network without altering the amorphous structure, as supported by TEM, XRD, and FTIR. Furthermore, zeta potential measurements showed a shift toward more positive values upon Ce and Zn doping, indicating partial neutralization of Si-OH groups and successful cation incorporation.

N_2_ adsorption-desorption isotherms were employed to characterize the pore structure of xCe-yZn-Sr-MBGNs, including specific surface area, pore diameter, and pore volume (Figure 3c, Table 2). All samples exhibited similar hysteresis loops, indicating that Ce, Zn, and Sr doping did not alter the mesoporous characteristics. The isotherms displayed a rounded knee and hysteresis loop within a relative pressure range of 0.45–0.9 P/P_0_, consistent with a Type IV isotherm, confirming a mesoporous structure [34]. The rounded knee reflects monolayer adsorption, while the hysteresis loop corresponds to capillary condensation within the pores [35]. Mesopores enhance the surface-to-volume ratio, specific surface area, and the capacity to load molecules for therapeutic applications [36]. These findings align with previous reports on CTAB-templated MBGNs and Ce- or Cu-doped MBGNs, which also exhibited Type IV isotherms regardless of cation incorporation [15,20,23]. Textural analysis revealed comparable specific surface areas for 0Ce-0Zn-Sr-MBGNs, 1Ce-0Zn-Sr-MBGNs, and 2Ce-0Zn-Sr-MBGNs (372.5, 423.4, and 377.2 m^2^/g, respectively). Similarly, Zn doping in 1Ce-yZn-Sr-MBGNs and 2Ce-yZn-Sr-MBGNs did not significantly affect surface area. Average pore diameters ranged from 9.2 to 12.0 nm, consistent with mesoporous structures (2–50 nm) [7], and pore volumes were comparable (16.1–17.3 cm^3^/g), irrespective of Ce or Zn content. These results indicate that incorporating Ce during the sol-gel process and cation doping via post-functionalization preserved the specific surface area and mesoporous structure. Since surface area directly influences particle degradation and ion release, all samples are expected to exhibit similar ion release profiles, thereby supporting comparable cellular activity [37].

### 2.2. Biodegradation and Ion Release Profile

**Figure 4:**
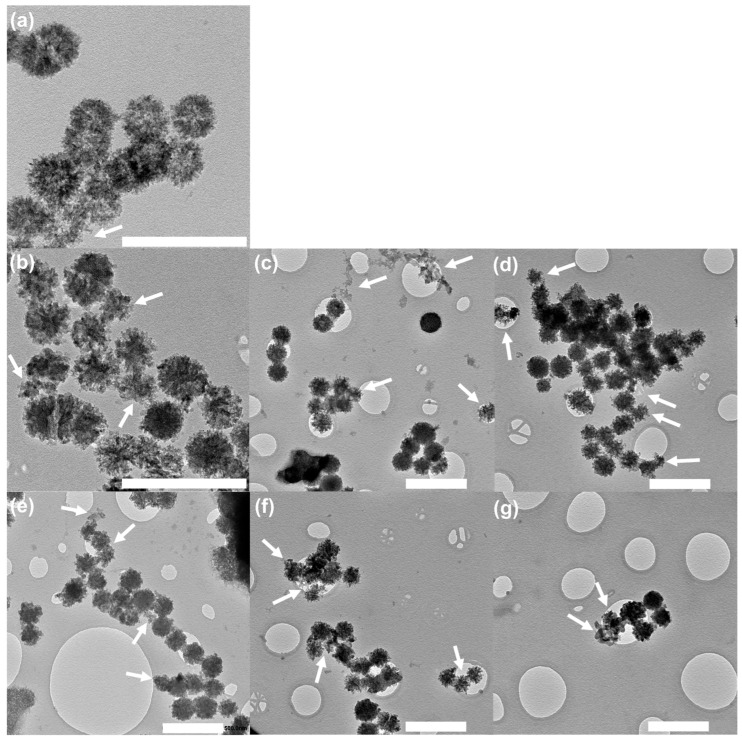
TEM images of xCe-yZn-Sr-MBGNs (**a**) 0Ce-0Zn-Sr-MBGNs, (**b**) 1Ce-0Zn-Sr-MBGNs, (**c**) 1Ce-0.5Zn-Sr-MBGNs, (**d**) 1Ce-1Zn-Sr-MBGNs, (**e**) 2Ce-0Zn-Sr-MBGNs, (**f**) 2Ce-0.5Zn-Sr-MBGNs, and (**g**) 2Ce-1Zn-Sr-MBGNs after soaking in PBS for 21 days. White arrows indicated degradation of the particles. Scale bar = 500 nm.

**Figure 5:**
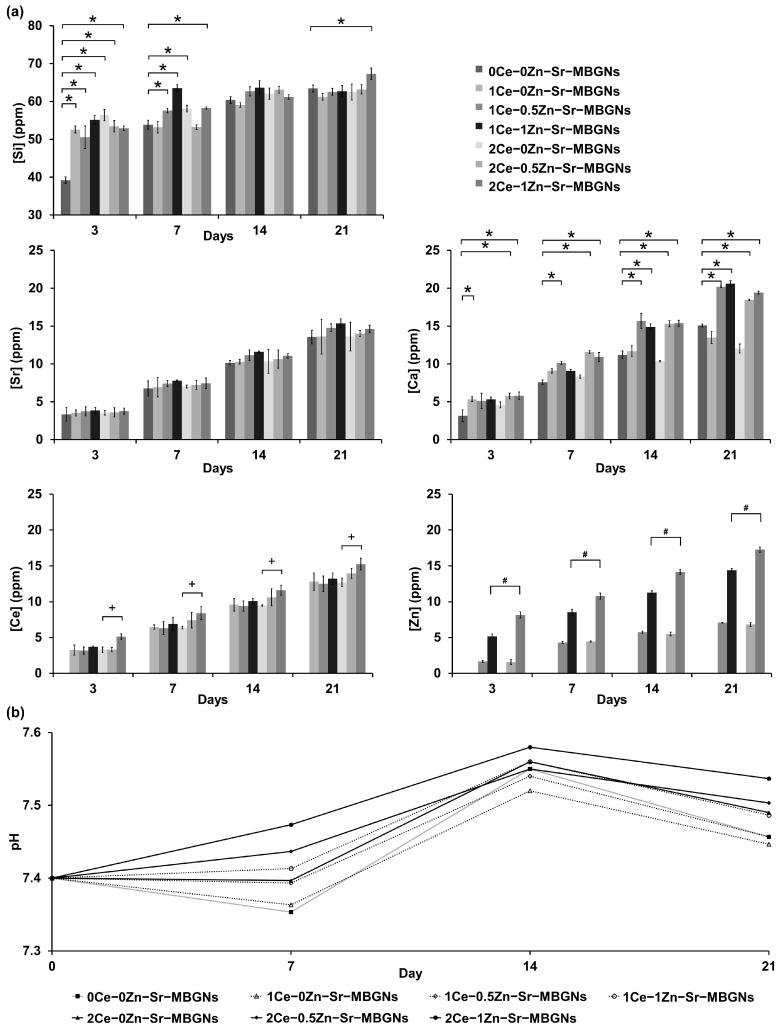
Ion release profiles and pH variation of xCe–yZn–Sr–MBGNs during incubation. (**a**) Concentrations of Si, Sr, Ca, Ce, and Zn ions released at 3, 7, 14, and 21 days. (**b**) pH variation of the immersion medium measured at 0, 7, 14, and 21 days. Data are presented as mean ± standard deviation (SD, *n* = 3). Statistical significance at each time point (*p* < 0.05) is indicated by symbols above the brackets as follows: (*) comparison with the control group (0Ce–0Zn–Sr–MBGNs); (+) comparisons among samples with the same Ce content; and (#) comparisons among samples with the same Zn content. Brackets denote pairwise comparisons between groups.

The amorphous structure confirmed by XRD, together with the elemental analysis determined by EDS, plays a pivotal role in dictating the degradability and ion release behavior of the particles. To further elucidate this relationship, degradation and ion release were investigated using TEM and ICP-OES. TEM images (Figure 4) revealed pronounced morphological degradation of xCe-yZn-Sr-MBGNs after 21 days in PBS, with particles exhibiting irregular and fragmented structures. The degradation of xCe-yZn-Sr-MBGNs followed a surface-initiated dissolution process governed by ion exchange and the hydrolysis of the silicate network. Upon immersion in PBS, modifier cations such as Sr, Zn, and Ce were rapidly exchanged with H^+^/H_3_O^+^, thereby weakening the Si-O-Si framework. This is followed by progressive hydrolysis of the silica backbone, leading to the gradual erosion of the outer mesoporous shell and the subsequent enlargement or collapse of the internal pore channels [38,39]. As degradation proceeded, the internal structure became hollowed and fragmented into smaller silicate clusters, as observed in the TEM analysis (Figure 4). Ce-0Zn-Sr-MBGNs retained a relatively regular morphology, suggesting that both Zn and Ce doping accelerate degradation. This observation showed reduced Si content with increasing Ce incorporation, suggesting a weakened silica network. Furthermore, Zn-doped nanoparticles exhibited more irregular, rough-edged morphologies compared to Zn-free samples, which is consistent with the role of Zn as a network modifier that disrupts the glass structure and enhances disorder. These morphological changes correlate well with the ion release profiles measured by ICP-OES (Figure 5), confirming the strong link between network composition, structural integrity, and degradation behavior.

The release profiles of Si, Ca, Sr, Ce, and Zn from xCe-yZn-Sr-MBGNs are presented in Figure 5a. Ions exhibited sustained release over 21 days in simulated physiological conditions, with distinct trends influenced by Ce and Zn incorporation. The ions exhibited sustained, time-dependent release, with characteristic concentration profiles and dissolution behaviors indicative of their specific functions as network dopants within the silica network. The pH variation in the incubation medium containing xCe-yZn-Sr-MBGNs was monitored over 7, 14, and 21 days to evaluate the degradation behavior and ion exchange process of the nanoparticles (Figure 5b). All samples stabilized within the physiological range (7.36–7.58) over 21 days. These findings were consistent with previous reports indicating pH elevation in ZnO-doped bioactive glasses due to cation release [40]. In this study, all particles showed sustained Si release. Within 3 days, 0Ce-0Zn-Sr-MBGNs released ~40 ppm Si compared to 50–56 ppm from Ce- and Zn-doped samples. This difference reflects the markedly higher Si content in 0Ce-0Zn-Sr-MBGNs (∼90 mol%) versus 1Ce-yZn-Sr-MBGNs (∼83 mol%) and 2Ce-yZn-Sr-MBGNs (∼74 mol%). Thus, the lower concentration of network modifiers in 0Ce-0Zn-Sr-MBGNs yields a more stable silica structure and slower Si release, consistent with previous findings [17].

Both Sr and Ca exhibited a time-dependent release profile, characterized by gradual and sustained ion dissolution without evidence of burst release. The sustained release reduced the risk of cytotoxic concentrations and ensured long-term bioactivity. For Sr, the release concentration progressively increased with immersion time, reaching ~15 ppm at 21 days. Ca release showed a similar increasing trend, achieving concentrations of ~20 ppm at 21 days. Ca release levels were consistently higher than Sr across all compositions, likely due to the higher initial Ca composition. Among the different compositions, Ce- and Zn-containing MBGNs (1Ce-1Zn-Sr-MBGNs) exhibited the highest cumulative Ca and Sr release, while Ce-only or Zn-only modifications yielded intermediate profiles. This may be attributed to multiple therapeutic effects of Ce and Zn on the glass structure, where Ce acts as a redox-active stabilizer influencing dissolution kinetics, while Zn serves as a partial network modifier, both collectively weakening the silicate framework and promoting ion release. These are consistent with previous reports where the incorporation of network-modifying cations enhanced the dissolution kinetics of MBGNs, thereby promoting therapeutic ion delivery [41]. The sustained release of Sr and Ca ions was crucial for bone regeneration. Sr promoted osteoblast activity while inhibiting osteoclasts, exerting dual anabolic and antiresorptive effects [12,42]. Ca supported hydroxyapatite nucleation, mineralization, and osteogenic signaling [43,44]. Their co-release from Ce- and Zn-doped MBGNs thus created a favorable ionic environment for bone repair.

For Ce, a continued release was observed, increasing from ~3 ppm on day 3 to ~20 ppm on day 21. The absence of a burst release indicates that a comparable release behavior was reported previously, showing a sustained Ce release of approximately 4 ppm over 72 h [20]. The observed discrepancy in the present study may be attributed to the incorporation of Ce into the glass network as nanoceria. Owing to its crystalline nature, nanoceria possesses higher structural stability, which restricts its dissolution and subsequent ion release [45]. The higher cumulative Ce release in 2Ce-doped MBGNs compared with 1Ce-doped samples further supports the dopant-concentration-dependent release. The Ce sustained release implied the potential for prolonged modulation of oxidative stress in the local tissue environment, an important consideration for bone regeneration and inflammation control. Zn release showed a more complex pattern, with concentrations ranging from ~2 ppm at day 3 to as high as ~18 ppm at day 21 in Zn-containing formulations. Notably, Zn release was highly composition-dependent, with xCe-1Zn-Sr-MBGNs displaying the highest overall release, while samples with lower Zn doping (xCe-0.5Zn-Sr-MBGNs) exhibited lower levels. Elevated Zn release was consistent with its role as a network modifier, which locally disrupts silicate bonds and enhances ion mobility. The concentrations fell within the reported therapeutic window for osteogenic stimulation, where Zn enhances osteoblast differentiation, collagen synthesis, and alkaline phosphatase activity, while also exerting antibacterial effects [46,47]. The co-doping strategy appeared to generate multifunctional effects, with Ce stabilizing the glass network while Zn enhances its degradability, resulting in tailored ion release profiles. Overall, the composition of xCe-yZn-Sr-MBGNs directly affected their degradability and dissolution behavior. Network-modifying cations accelerate degradation by disrupting the silica framework, whereas higher Si content stabilizes the network and slows dissolution [48].

The sustained release profiles indicated a continuous degradation mechanism of the xCe-yZn-Sr-MBGNs. The release kinetics further indicated a tunable system, where doping levels can balance structural stability with therapeutic ion delivery. In summary, the incorporation of Ce and Zn dopants modulate ion release, producing sustained, composition-dependent profiles that augment the multifunctional potential of MBGNs in the context of bone regeneration. Collectively, the regulated release of ions from MBGNs implies that the material has the capacity to create a conducive ionic microenvironment that supports bone regeneration while concurrently alleviating oxidative stress and reducing infection risks. The adaptability of release profiles through dopant modulation underscores the versatility of this system for tailored therapeutic strategies.

### 2.3. In Vitro Bioactivity

**Figure 6:**
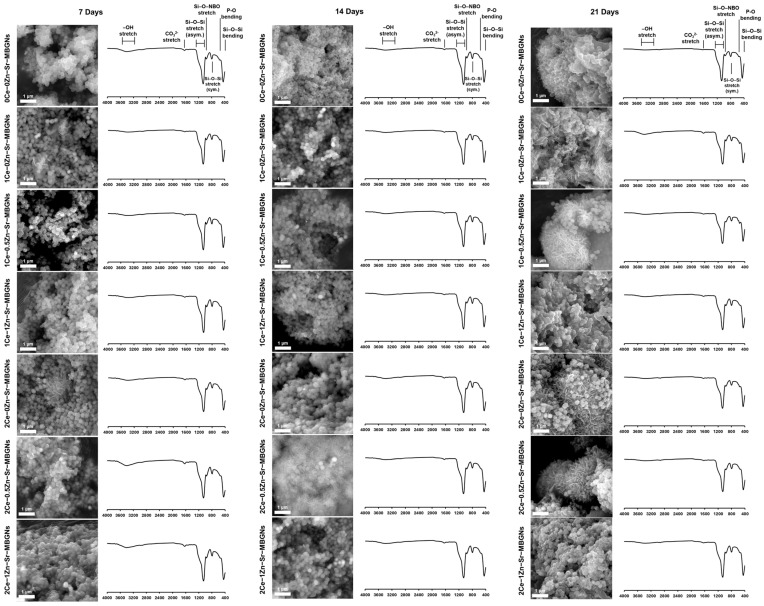
The SEM image and FTIR spectra of xCe-yZn-Sr-MBGNs after being immersed in SBF for 7, 14, and 21 days. Scale bar = 1 μm.

The in vitro bioactivity of xCe-yZn-Sr-MBGNs was evaluated by assessing their ability to form a hydroxycarbonate apatite (HCAp) layer, which is essential for bonding with surrounding tissue and supporting bone cell growth. The bioactivity of xCe-yZn-Sr-MBGNs was assessed through apatite formation in the SBF solution. XRD confirmed their amorphous nature, which facilitates ion release and degradation, supporting bioactivity. The released ions facilitated exchange with simulated body fluid (SBF), thereby triggering apatite formation and enhancing particle bioactivity. The surface morphology of the xCe-yZn-Sr-MBGNs after immersion in the SBF solution was examined using SEM (Figure 6). Apatite formation was first observed after 7 days of immersion in the SBF. Needle-shaped crystalline structures were prominently observed in 2Ce-0.5Zn-Sr-MBGNs on day 14, whereas they significantly appeared in other samples by day 21. In contrast, 1Ce-1Zn-Sr-MBGNs and 2Ce-1Zn-Sr-MBGNs exhibited less apatite formation. Specifically, Zn^2+^ ions were known to adsorb onto the active growth sites of HCAp nuclei, thereby poisoning the crystal growth and favoring the formation of stable, amorphous zinc phosphate layers Similarly, the high affinity of Ce for phosphate groups can lead to the formation of CePO_4_, which possesses a lower solubility product than HCAp, effectively consuming the available phosphate and retarding apatite nucleation [15,48,49]. Ca was essential for forming the CaP domain, which transitions from an amorphous CaO-P_2_O_5_-rich (ACP) layer into crystalline HCAp. ICP-OES revealed low Ca ion release across all samples, which may explain the delayed apatite formation compared with previous studies [50,51]. The slow dissolution of the silicate network, likely stabilized by the presence of Ce and Sr, limits the supersaturation of Ca^2+^ and PO_4_^3−^ ions at the glass-solution interface, extending the induction period for HCAp crystallization. FTIR spectra confirmed bioactivity by showing characteristic bands of hydroxyl, carbonate, and phosphate groups. The phosphate bands (500–600 cm^−1^) intensified with immersion time, indicating the transition from amorphous phosphate to a crystalline HCAp phase [52]. Carbonate substitution, identified at 1600–1400 cm^−1^, further confirmed the presence of HCAp [15,48,53]. Overall, while all samples exhibited bioactivity, apatite formation was delayed in Zn- and Ce-rich compositions due to competitive phosphate interactions and low Ca availability.

### 2.4. DPPH Radical Scavenging Assay

**Figure 7:**
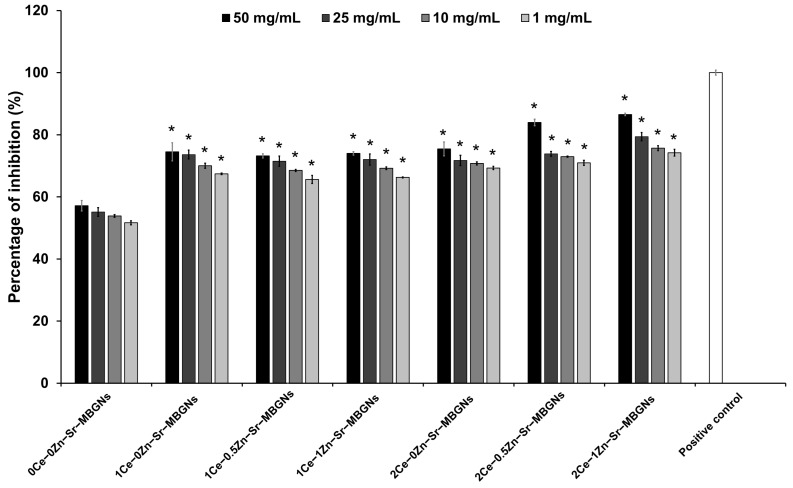
The %inhibition after incubation of each type of particle with DPPH. (*n* = 3). A statistically significant difference compared to 0Ce-0Zn-Sr-MBGNs at the same concentration was indicated by * *p* < 0.05. Statistical analysis was performed using one-way ANOVA followed by Tukey’s post hoc test. Ascorbic acid served as the positive control, represented by the white bar.

The antioxidant activity of xCe-yZn-Sr-MBGNs was evaluated using the DPPH radical scavenging assay, which monitors the conversion of the purple DPPH radical to its non-radical yellow form (DPPH-H) [54]. As shown in Figure 7, DPPH inhibition increased in both a concentration- and Ce- and Zn-content-dependent manner, with higher activity observed at 50 mg/mL, particularly in 2Ce-0.5Zn-Sr-MBGNs and 2Ce-1Zn-Sr-MBGNs. Co-doping with Zn further enhanced antioxidant activity compared to Ce-only samples, indicating an additional effect. Zn likely promotes redox activity by increasing oxygen vacancies and electron mobility within the lattice, facilitating more efficient Ce^3+^/Ce^4+^ redox cycling. Notably, DPPH scavenging was slower than in previous studies [55,56], likely due to nanoceria being embedded within the porous MBGN structure. Interestingly, 2Ce-0Zn-Sr-MBGNs showed higher DPPH inhibition than 1Ce-0Zn-Sr-MBGNs, likely due to their higher Ce content, emphasizing Ce’s central role in radical scavenging [57]. In summary, MBGNs containing nanoceria exhibit robust antioxidant activity, which is further enhanced by Zn co-doping, demonstrating an additional effect between Ce and Zn in promoting radical scavenging efficiency. While the DPPH assay effectively demonstrated the intrinsic chemical radical scavenging capacity of the xCe-yZn-Sr-MBGNs, the current study was limited to abiotic antioxidant evaluation. The direct assessment of intracellular ROS levels and the subsequent signaling pathways within cells remain an important area for further investigation.

### 2.5. In Vitro Cytotoxicity

**Figure 8:**
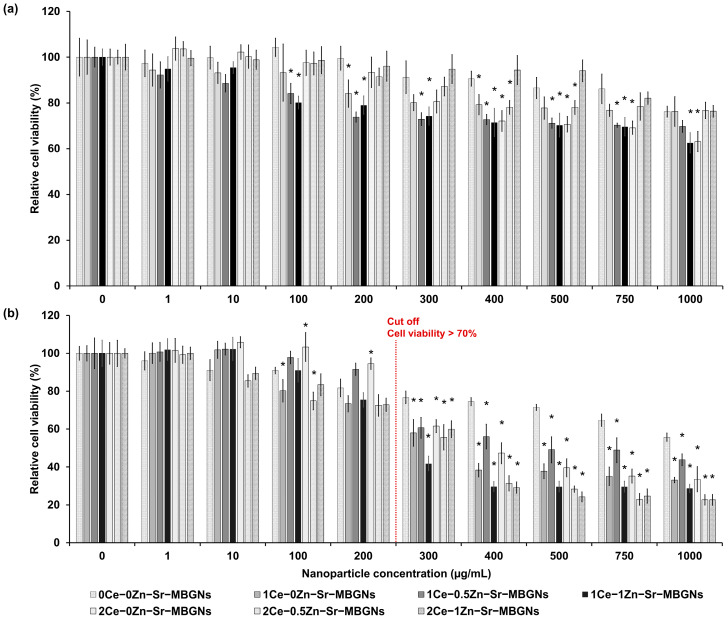
Relative cell viability of MC3T3-E1 cells (MTT assay) cultured directly in contact with particles at a range of 0–1000 µg/mL for (**a**) 24 h and (**b**) 72 h. Data were normalized with respect to the control (cells cultured in a basal medium), expressed as mean ± SD (*n* = 6) * indicates a statistically significant difference compared to the negative control (cells cultured in a basal medium) at each interval concentration (*p*-value < 0.05). The red dashed line indicates the cutoff level corresponding to 70% cell viability.

The cytotoxicity of xCe-yZn-Sr-MBGNs was evaluated using the MTT assay on MC3T3-E1 cells at concentrations ranging from 0 to 1000 μg/mL after 24 and 72 h of exposure. The systematic variation in concentrations of Ce (x: 0, 1, 2 mol%) and Zn (y: 0, 0.5, 1 mol%) was designed to map the biological response across a potential therapeutic gradient. Untreated cells served as the control. Cell viability below 70% was considered cytotoxic [58]. The results showed that none of the samples exhibited cytotoxicity within the first 24 h at concentrations up to 750 μg/mL (Figure 8a). However, after 72 h, all samples reduced MC3T3-E1 cell viability in a time-dependent manner (Figure 8b). Particles containing Ce and Zn exhibited greater cytotoxicity than undoped 0Ce-0Zn-Sr-MBGNs, with toxic effects observed above 200 μg/mL. Notably, Ce-doped samples reduced viability at concentrations ≥ 200 μg/mL, whereas 0Ce-0Zn-Sr-MBGNs remained non-toxic up to 500 μg/mL. Increased Ce content further exacerbated cytotoxicity, particularly in 1Ce-1Zn-Sr-MBGNs and 2Ce-1Zn-Sr-MBGNs. In contrast, no cytotoxicity up to 300 μg/mL for Ce-MBGNs containing 5–10 mol% Ce was reported after 72 h and 100 μg/mL for 1 mol% Ce-MBGNs after 48 h [16,20,59]. The discrepancy with the present findings may result from higher actual Ce incorporation than the calculated values, as confirmed by XRF, leading to stronger toxic responses. Excess Ce can disrupt mitochondrial membrane integrity, generate ROS, and cause DNA fragmentation, resulting in reduced cell viability [60]. Zn content also influenced cytotoxicity. At comparable Ce levels, 1Ce-0.5Zn-Sr-MBGNs and 1Ce-1Zn-Sr-MBGNs showed greater toxicity, consistent with Zn’s dual role as a pro- or anti-apoptotic agent. At high doses, Zn can trigger apoptosis via activation of p38, modulation of potassium channels, and disruption of metabolic pathways [61]. Together, these results demonstrate that cytotoxicity is predominantly governed by Ce concentration, with Zn serving as a secondary contributor. Based on these findings, 200 μg/mL was selected as the threshold concentration for subsequent osteogenic differentiation analysis.

### 2.6. Osteogenic Differentiation

#### 2.6.1. Calcify Formation

**Figure 9:**
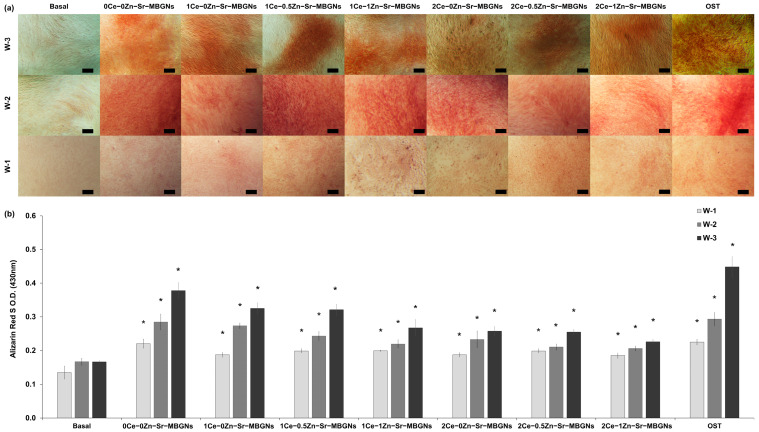
Alizarin Red S staining (**a**) calcified nodule formation from MC3T3-E1 cells treated with xCe-yZn-Sr-MBGNs (a concentration of 200 mg/mL) for 3 weeks using inverted light microscopy (LABOMED TCM400, Los Angeles, CA, USA) at magnification 4×, scale bar = 200 µm. (**b**) Semi-quantification of calcium deposits of MC3T3-E1 cells (*n* = 4). * indicates a statistically significant difference compared to the negative control (cells cultured in a basal medium) at each interval time (*p* < 0.05). W-1, W-2, and W-3 represent weeks 1, 2, and 3, respectively.

Calcium deposition, a marker of osteoblast differentiation and late-stage mineralization, was evaluated in MC3T3-E1 cells cultured with xCe-yZn-Sr-MBGNs. Alizarin Red S staining confirmed the calcium nodule formation in all treated groups (Figure 9). Cells exposed to xCe-yZn-Sr-MBGNs exhibited markedly greater, red-stained areas compared to cells maintained under basal conditions, with significant increases observed at both day 14 and day 21 (Figure 9a). Semi-quantitative analysis further revealed a statistically significant elevation in calcium deposition in treated groups relative to untreated controls at each time point, confirming the mineralization-promoting effect of the particles (Figure 9b). This outcome correlates with the bioactivity results, which confirmed apatite-like crystal formation on particle surfaces, further supporting the mineralization potential of these glasses.

Among the formulated compositions, 0Ce-0Zn-Sr-MBGNs induced the highest level of calcium deposition, which can be attributed to the sustained release of essential osteogenic ions (Si, Ca, and Sr), thereby providing a greater supply of critical components required for matrix mineralization [62]. However, increasing Ce content resulted in a reduction in mineralization, as observed in 1Ce-yZn-Sr-MBGNs and 2Ce-yZn-Sr-MBGNs. This discrepancy may result from excessive Ce incorporation, which disrupts redox balance and mitochondrial function, thereby hindering osteoblast differentiation. The heightened redox activity of Ce-containing particles observed in nitrite assays supports this interpretation, and the formation of insoluble CePO_4_ may further contribute to this [63]. Similar inhibitory effects of high Ce concentrations on osteogenesis have been reported, aligning with the present findings [64,65]. In addition, increasing Zn content (xCe-1Zn-Sr-MBGNs) led to a reduction in mineralization compared with samples containing lower Zn levels (xCe-0.5Zn-Sr-MBGNs) or no Zn (xCe-0Zn-Sr-MBGNs). This limited response resulted from the relatively low Zn content combined with the inhibitory effect of Ce. Taken together, these findings suggested a composition-dependent balance between Ce- and Zn-induced effects on osteoblast mineralization. While a moderate concentration of zinc doping facilitates osteogenic activity, an excessive presence of cerium appears to adversely affect this process, potentially negating the advantageous effects associated with the release of Si, Ca, and Sr. These results emphasize the critical need for optimizing the ratios of Ce and Zn in xCe-yZn-Sr-MBGNs to attain optimal osteogenic responses and highlight the complex interplay between the release of therapeutic ions, redox modulation, and signaling pathways within the domain of bone tissue engineering.

#### 2.6.2. Osteogenic Gene Expression

**Figure 10:**
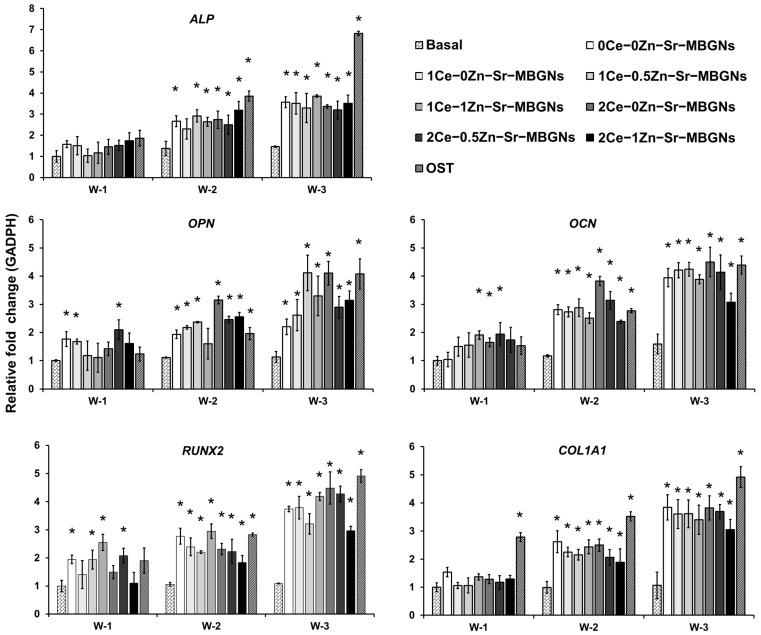
Quantitative real-time reverse transcriptase PCR analysis showing the expression of osteogenic marker genes in MC3T3-E1 cells treated with xCe-yZn-Sr-MBGNs, cultured over 3 weeks. W-1, W-2, and W-3 represent weeks 1, 2, and 3, respectively. Gene expression was normalized to *GAPDH* (a housekeeping gene) transcript levels. The data represents two independent experiments. The values are the mean ± SD, *n* = 3. The expression of osteogenic marker genes was significantly upregulated at the indicated times in cells treated with particles compared to untreated cells. * above the bars represent a significant difference between treated MC3T3-E1 versus untreated MC3T3-E1 under the basal condition (*p* < 0.05).

The osteogenic differentiation of pre-osteoblastic cells exposed to xCe-yZn-Sr-MBGNs was evaluated through quantitative reverse transcription polymerase chain reaction (qRT-PCR), focusing on early- (*ALP, RUNX2*), intermediate- (*COL1A1, OPN*), and late-stage (*OCN*) biomarkers [66,67] over a duration of three weeks (Figure 10). *ALP* expression, an early osteoblastic marker, increased progressively with culture duration in all treated groups compared to the untreated group, with the most significant upregulation observed at weeks 2 and 3, suggesting that the incorporation of Ce and Zn stimulated early osteogenic commitment. *COL1A1* expression, a key extracellular matrix gene, was upregulated from week 2 onwards, with sustained elevation through week 3, indicating that the co-substitution of Ce and Zn enhanced matrix deposition. *RUNX2* expression, a master transcription factor regulating osteogenesis, was consistently upregulated throughout the study period, demonstrating the additive effects of Ce and Zn in the activation of osteogenic signaling pathways. *OPN* and *OCN* expressions, representing intermediate and late differentiation, respectively, demonstrated time-dependent increases across all treated groups. *OPN* expression was significantly enhanced at week 3, whereas *OCN* expression reached comparable levels across most nanoparticle groups, indicating effective maturation of osteoblasts.

Although the osteogenic supplement (OST) group displayed the highest expression overall, cells exposed to xCe-yZn-Sr-MBGNs achieved nearly comparable levels in the absence of external osteogenic supplementation, highlighting the intrinsic osteoinductive capability of these compositions. The gene expression results demonstrate that xCe-yZn-Sr-MBGNs influence osteogenic differentiation through a coordinated, stage-dependent mechanistic framework. Upregulation of *ALP* and *RUNX2* at early time points suggested that an appropriate balance of Ce and Zn promotes osteoblastic commitment via specific intracellular pathways. Specifically, the concurrent release of Sr is likely to activate the Ca-sensing receptor (CaSR), further enhancing osteogenic differentiation [68,69]. The sustained release of Sr and Ca from the mesoporous framework acts as an extracellular signal that triggers the G-protein-coupled CaSR, leading to the activation of downstream transcription factors essential for bone formation [70]. Furthermore, the role of Zn when released from the particles extends beyond its enzymatic function as a cofactor for ALP. Zn ions are known to stabilize the Zinc finger domains within various transcription factors, which are critical for DNA binding and the transcriptional activation of osteogenic genes [71]. The transition from early commitment to matrix maturation is evidenced by the sustained expression of *COL1A1*, *OPN*, and *OCN*. During the high metabolic activity associated with matrix synthesis and mineral deposition, cells naturally produce higher levels of ROS. Excessive ROS can lead to mitochondrial dysfunction and the inhibition of osteoblast maturation. At optimal formulation (1Ce-1Zn-Sr-MBGNs), the presence of nanoceria within the xCe-yZn-Sr-MBGN structure provides an in situ enzymatic defense. By alternating between Ce^3+^/Ce^4+^ states, the nanoparticles mimic Superoxide Dismutase (SOD) and Catalase, effectively quenching ROS [72]. This antioxidant shield ensures that the signaling pathways activated by Sr and Zn remain uninterrupted, allowing for the observed time-dependent increase in *OCN* expression and the subsequent formation of a mineralized apatite layer. However, excessive incorporation of Ce and Zn, as in 2Ce-1Zn-Sr-MBGNs, was associated with reduced expression of osteoblastic markers. This suggests that elevated Ce and Zn concentrations may disrupt mitochondrial activity and interfere with phosphate metabolism, creating a redox imbalance that hinders differentiation [19]. Taken together, these results highlight a complementary ionic orchestration. Sr primarily supports mineral deposition, Zn enhances ALP activity and *RUNX2* expression via MAPK pathways, and Ce provides essential redox modulation. These findings underscore the fact that Ce exerts its effects within a narrow therapeutic range, requiring precise compositional control to optimize osteogenic performance while minimizing adverse cellular responses.

### 2.7. Cell Migration

**Figure 11:**
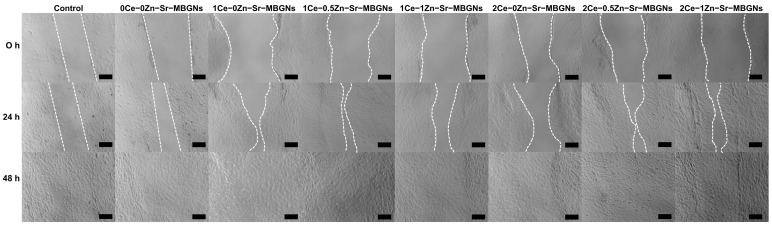
In vitro scratch assay of MC3T3-E1 cells. A scratch cell migration assay was conducted with MC3T3-E1 cells treated with xCe-yZn-Sr-MBGNs. Cells cultured in a basal medium without particles served as the control. Phase-contrast microscopic images were captured at 0, 24, and 48 h after scratching using inverted light microscopy at magnification 4×, scale bar = 200 µm.

To investigate the impact of xCe-yZn-Sr-MBGNs on the biological function of MC3T3-E1 cells, an in vitro scratch assay was performed. In this study, MC3T3-E1 cells were pre-treated with xCe-yZn-Sr-MBGNs for 48 h prior to the scratch assay, rather than being maintained in a particle-supplemented medium during the migration phase. This approach was specifically chosen to evaluate the sustained biological modifications and cellular priming induced by nanoparticle uptake independently of any immediate physical interference. By removing the particles and providing fresh medium at the time of the scratch, the observed migration rates (0, 24, and 48 h) more accurately reflect the intrinsic migratory capacity of the cells as a direct result of their prior interaction with the nanoparticles. The results of the scratch assay indicated that the wound healing rate in the group treated with 200 µg/mL xCe-yZn-Sr-MBGNs was significantly higher than that of the control group in 24 h after scratching (Figure 11).

Approximately 70% of the scratch area was covered by migrated cells within 24 h in all treatment groups, except for the 2Ce-0Zn-Sr-MBGNs group, where only about 25% of the gap was filled within the same period. After 48 h, the wound gap was nearly closed in all groups. Interestingly, cells treated with xCe-yZn-Sr-MBGNs exhibited a faster migration rate compared to the control group, suggesting that the therapeutic ions released from these particles activated the biological functions of MC3T3-E1 cells. This finding was consistent with previous studies reporting that MBGNs stimulated the migration and motility of early pre-osteoblastic cells compared to untreated cells [73]. The enhanced migration observed here may be attributed to additive effects of Si, Ca, Ce, Zn, and Sr ions, which could modulate signaling pathways related to cytoskeletal organization and proliferation. Faster cell migration is critical in bone tissue regeneration, as it facilitates rapid coverage of bone defects and promotes subsequent osteogenic differentiation.

### 2.8. Antibacterial Activity

**Figure 12:**
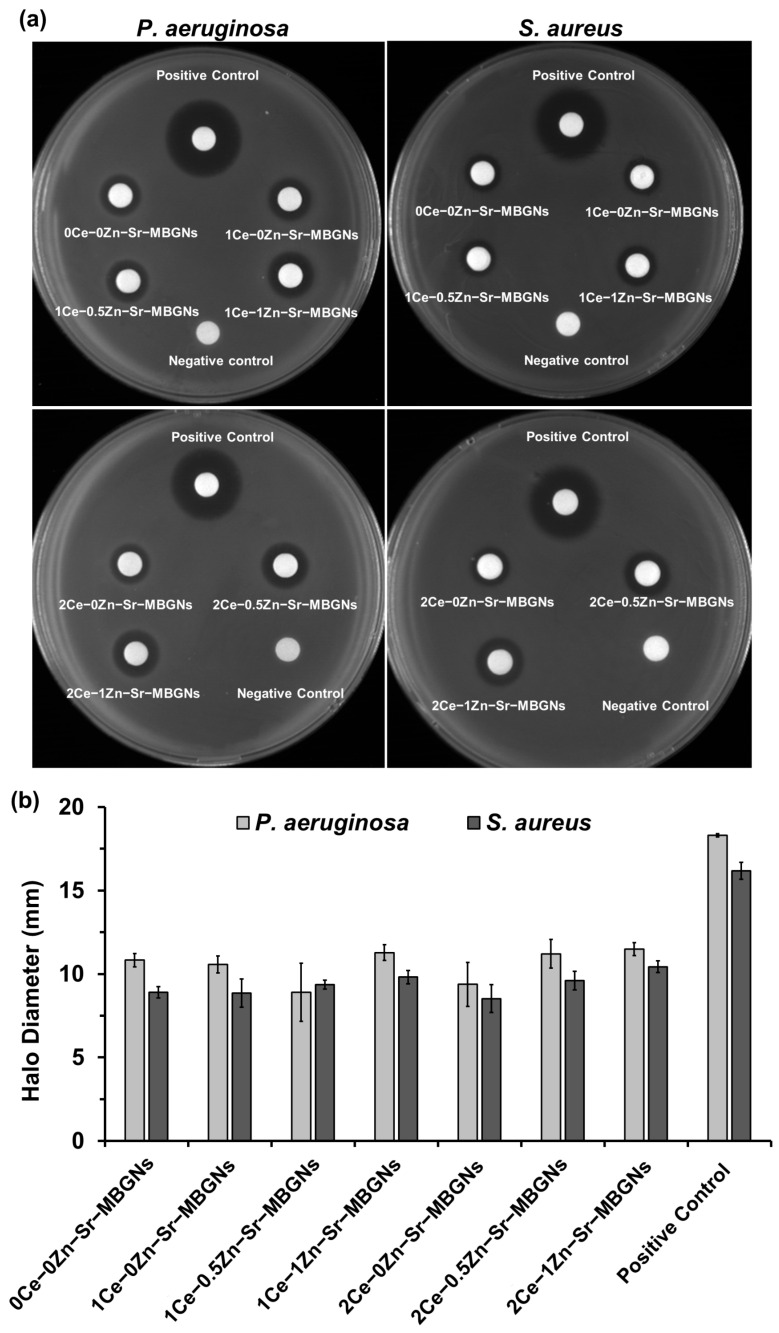
In vitro antibacterial activity of samples against *P. aeruginosa* and *S. aureus* after incubation for 18 h. (**a**) Zone of inhibition from the disk diffusion assay. (**b**) Diameter of the clear zone. The values are the mean ± SD, *n* = 3.

The preliminary antibacterial potential of the MBGNs was confirmed using the disk diffusion assay against Gram-negative (*P. aeruginosa*) and Gram-positive (*S. aureus*) bacteria. *P. aeruginosa*, a common multidrug-resistant pathogen in hospital settings, was selected due to its clinical relevance [74]. The nanoparticle concentration at 10 mg/mL was applied in the disk diffusion assay. A concentration of 10 mg/mL was utilized to ensure sufficient interaction between the nanoparticles and the bacterial cell walls within the localized environment of a 6 mm disk. This dosage was specifically selected to account for the diffusion kinetics of the nanoparticles through the semi-solid agar matrix; unlike soluble antibiotics, solid-phase nanoparticles often exhibit limited mobility within the agar, necessitating a higher loading concentration to establish a detectable inhibitory gradient. While this concentration is higher than the dosages evaluated in cytotoxicity assays against MC3T3-E1 pre-osteoblast cells, the discrepancy is justified by the fundamental differences between the experimental platforms. In cell culture models, nanoparticles are in direct, prolonged contact with the cell monolayer in a liquid medium, where lower doses are sufficient to elicit a biological response. In contrast, the antibacterial disk assay requires a robust reservoir of bioactive material to overcome the physical resistance of agar and effectively disrupt the rapid proliferation of *S. aureus* and *P. aeruginosa*. The larger clear zone (halo diameter) refers to the greater ability to be antibacterial. In this study, penicillin–streptomycin was used as a positive control, and PBS was used as the negative control. The results demonstrate that doping xCe-yZn-Sr-MBGNs with Ce and Zn resulted in significant antibacterial properties. As shown in Figure 12, clear zones of inhibition were observed after 18 h of incubation against both Gram-negative (*P. aeruginosa*) and Gram-positive (*S. aureus*) bacteria, indicating effective antibacterial activity. These findings revealed reduced bacterial viability upon treatment with ion-doped particles. The observed antibacterial effect was likely attributed to the release of therapeutic ions from the particles, as evidenced by the ion release profiles and the presence of nanoceria, confirmed through ICO-OES, TEM, and XRD analyses. These components play a crucial role in enhancing the antibacterial performance of xCe-yZn-Sr-MBGNs. Undoped particles (0Ce-0Zn-Sr-MBGNs) exhibited minimal antibacterial effects, whereas co-doped particles (1Ce-0.5Zn-, 1Ce-1Zn-, 2Ce-0.5Zn-, and 2Ce-1Zn-Sr-MBGNs) showed increased activity, particularly against *S. aureus*, reflecting additive effects. This enhancement is likely mediated by the release of therapeutic ions and the presence of nanoceria, which can generate ROS and disrupt bacterial membranes [16]. Zn ions further impair bacterial metabolism by interfering with nucleic acids and enzyme function [75]. *P. aeruginosa* displayed slightly larger inhibition zones than *S. aureus*, consistent with Gram-positive bacteria’s thicker peptidoglycan layer, which limits ion penetration. Bacterial defense mechanisms, including efflux pumps, may also mitigate metal ion toxicity [76,77]. Nanoceria disrupts membrane integrity and induces oxidative stress via Ce^3+^/Ce^4+^ redox cycling, impairing nutrient transport and metabolic processes [78,79].

The disk diffusion method revealed relatively small inhibition zones, which may be attributed to the limited diffusion of the MBGNs and their released ions through the agar matrix. Therefore, these results primarily indicate antibacterial activity rather than bactericidal action, as complete bacterial killing was not confirmed [80,81]. Furthermore, comparisons with the penicillin–streptomycin control should be interpreted cautiously, given differences in diffusion and molecular size [82]. In summary, co-doping with Ce and Zn clearly enhances the antibacterial potential of xCe-yZn-Sr-MBGNs. This enhancement is likely driven by the combined effects of sustained ion release and nanoceria-induced oxidative stress, with greater impact on both Gram-positive and Gram-negative bacteria. Future studies should incorporate clinically relevant antibacterial evaluations, including MIC/MBC broth dilution assays, bacterial adhesion and biofilm formation on coated surfaces, to fully validate their performance for clinical implant applications. Additionally, mechanistic investigations of ROS-mediated activity and in vivo bone regeneration models will be critical in bridging the gap toward clinical translation.

## 3. Materials and Methods

All reagents were obtained from Sigma–Aldrich (St. Louis, MO, USA) unless stated otherwise. These include hexadecyltrimethylammonium bromide (CTAB, CH_3_(CH_2_)15N(Br)(CH_3_)_3_), tetraethyl orthosilicate (TEOS), ethyl acetate, absolute ethanol, 25% ammonia solution, calcium nitrate tetrahydrate (Ca(NO)_3_·4H_2_O), cerium (III) nitrate hexahydrate (Ce(NO_3_)_3_·6H_2_O), strontium nitrate (Sr(NO_3_)_2_), zinc nitrate tetrahydrate (Zn(NO_3_)_2_·4H_2_O), potassium chloride (KCl), di-potassium hydrogen phosphate trihydrate (K_2_HPO_4_.3H_2_O), magnesium chloride hexahydrate (MgCl_2_.6H_2_O), sodium hydrogen carbonate (Na-HCO_3_), sodium sulfate (Na_2_SO_4_), sodium chloride (NaCl), hydrochloric acid (HCl), toluene, calcium chloride (CaCl_2_), cetylpyridinium chloride, sodium phosphate, nitric acid, dimethyl sulfoxide (DMSO), dexamethasone (DEX), β-glycerophosphate, L-ascorbic acid, paraformaldehyde, Alizarin Red S. Cell culture reagents, including minimum essential medium eagle alpha (α-MEM), phosphate-buffered saline (PBS), Antibiotic-Antimycotic, Penicillin–Streptomycin, trypsin-EDTA, 3-(4,5-dimethylthiazol-2-yl)-2,5-diphenyltetrazolium bromide (MTT) were obtained from Gibco™, Thermo Fisher Scientific (Waltham, MA, USA). Mueller–Hinton Agar (MHA) and Mueller–Hinton Broth (MHB) were obtained from Difco™ (Bangkok, Thailand). Pre-osteoblast (MC3T3-E1: CRL-2593 ™) was purchased from Biomedia (Bangkok, Thailand). *Staphylococcus aureus* was obtained from the Department of Microbiology, Faculty of Science, King Mongkut’s University of Technology Thonburi, Thailand. *Pseudomonas aeruginosa* was obtained from the National Center for Genetic Engineering and Biotechnology, Pathum Thani, Thailand.

### 3.1. xCe-yZn-Sr-MBGNs Synthesis

**Figure 13:**
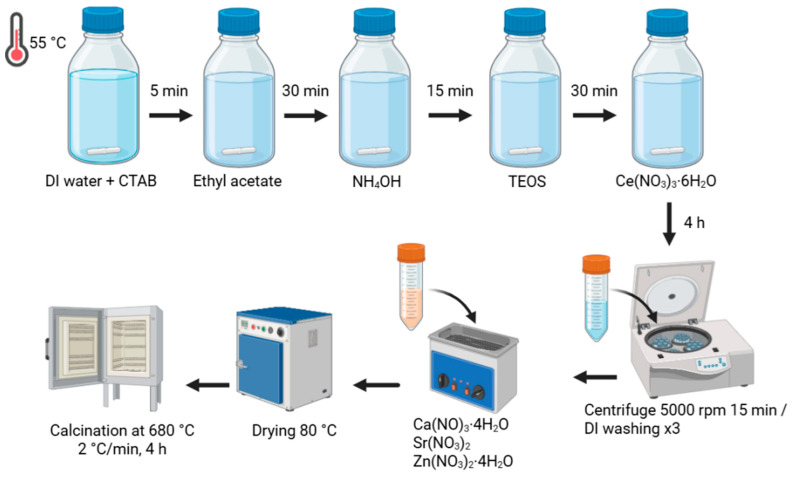
Preparation of xCe-yZn-Sr-MBGNs.

xCe-yZn-Sr-MBGNs were prepared using the microemulsion-assisted sol-gel method under basic conditions and post-functionalization (Figure 13). CTAB, a cation surfactant, was used to generate a pore structure. Nanoceria formed during the silica network formation and was subsequently trapped within the network. Mesoporous silica nanoparticles were first synthesized before Ca, Sr, and Zn incorporation through post-functionalization with various nominal rations (Table 3). Briefly, 273 mL of DI water was pre-heated until the temperature rose to 55 °C before adding CTAB 5.88 g at a stirring rate of 600 rpm for 5 min in a 1 L DURAN^®^ (DWK Life Sciences, Mainz, Germany). After that, 84 mL of ethyl acetate was added to the solution and mixed for 30 min. Then, 58.8 mL of 1 M ammonium hydroxide was added to the mixed solution at room temperature with a stirring rate of 600 rpm for 15 min. After that, 10 mL of TEOS was gently mixed and stirred for another 30 min. Ce(NO_3_)_3_·6H_2_O, a Ce precursor, was weighed with the nominal ratio (1Ce-yZn-Sr-MBGNs and 2Ce-yZn-Sr-MBGNs). The solution was mixed for 4 h to complete the hydrolysis and polycondensation reactions. The colloidal particles were collected using centrifugation at 5000 rpm for 30 min. The collected particles were washed twice with DI water to remove CTAB. Sr, Zn, and Ca were incorporated within the formed particles to extend the biological properties within the sonication bath for 1 h. The doped particles were collected using centrifugation at 5000 rpm for 30 min and washed twice with ethanol before being dried at 80 °C and calcined at 680 °C, with a heating rate of 2 °C/minute for 4 h to obtain xCe-yZn-Sr-MBGNs.

### 3.2. Characterization of xCe-yZn-Sr-MBGNs 

The size and morphology of xCe-yZn-Sr-MBGNs were detected using dynamic light scattering (DLS, Horiba SZ-100V2, Horiba, Kyoto, Japan) and field emission scanning electron microscopy with an accelerating voltage of 10 kV (FE-SEM, Phenom Pharos Desktop FEG SEM, ThermoFisher Scientific, Waltham, MA, USA). The particle size of the MBGNs observed in the SEM images was analyzed and validated using ImageJ software (version 1.41o, Java 1.6.0_10, Wayen Rasband, US National Institutes of Health, Bethesda, MD, USA). The nanoceria formation in 1Ce-yZn-Sr-MBGNs and 2Ce-yZn-Sr-MBGNs was investigated using the transmission electron microscope (TEM, JEM-2100 Plus, JEOL, Tokyo, Japan) with an accelerating voltage of 200 kV. The stability of particles in the DI water at pH 6.5 and a concentration of 1 mg/mL was measured using a Zeta sizer (Horiba SZ-100V2, Horiba, Kyoto, Japan). N2 adsorption/desorption isotherms were obtained using a surface area analyzer (BET, BELSORP-mini II, Bel Japan Inc., Osaka, Japan). The specific surface area, porous structure, pore diameter, and pore volume were determined by BET multipoint analysis, with P/Po data points selected within the range of 0.05–0.35. Before measurement, the samples were degassed at 200 °C for 24 h at a heating rate of 10 °C/min. The amorphous structure in the nature of the samples was confirmed using X-ray diffraction (XRD; Bruker AXS Model D8 Advance, Karlsruhe, Germany). XRD patterns were obtained using a Bruker AXS automated powder diffractometer equipped with Cu Kα radiation (λ = 1.5406 Å) operated at 40 kV/40 mA. Data were recorded over the 2θ range of 10–70°, with a step size of 0.02° and a dwell time of 0.5 s per step. The functional groups of the xCe-yZn-Sr-MBGNs were identified using Fourier transform infrared spectroscopy (FTIR; BRUKER IINVENIO-S, Bruker Optics GmbH & Co. KG, Ettlingen, Germany). The FTIR spectra were recorded in attenuated total reflection (ATR) mode over the wavenumber range of 2000–400 cm^−1^, with a resolution of 4 cm^−1^ and a scan speed of 32 scans/min.

### 3.3. Biodegradation and Ion Release Profile

For the ion release and biodegradation profiles, a sacrificial sampling approach was employed to ensure independent measurements at each time point. Briefly, 37.5 mg of xCe-yZn-Sr-MBGNs was immersed in 25 mL of PBS (pH 7.4) and incubated at 37 °C with constant shaking at 120 rpm. The degradation and the release profiles were monitored as a function of time for 21 days. At each predetermined time interval (3, 7, 14, and 21 days), specific sets of samples were removed from the incubator and processed. This sacrificial method was selected to mitigate potential errors associated with volume loss and concentration fluctuations common in repeated sampling. The particles were separated by centrifugation, followed by sequential washing with ethanol and acetone to terminate the reactions. The morphological change in the degraded particles at day 21 was investigated using the transmission electron microscope (TEM, JEM-2100 Plus, JEOL, Tokyo, Japan) with an accelerating voltage of 200 kV. The concentration of released ions (Si, Ca, Ce, Sr, and Zn) in the supernatants was analyzed using inductively coupled plasma optical emission spectroscopy (ICP-OES, iCAP 6000 series, Thermo Fisher Scientific, Cambridge, UK) to quantify the concentration of released ions. All experiments were performed in triplicate (*n* = 3).

### 3.4. In Vitro Bioactivity

In vitro bioactivity was evaluated to demonstrate the ability of xCe-yZn-Sr-MBGNs to induce hydroxyapatite layer formation, which serves as a favorable surface for bone cell bonding by incubating the particles in simulated body fluid (SBF) solution at pH 7.4 and 37 °C for 7, 14, and 21 days. The SBF solution was prepared following a previous study [83]. In total, 37.5 mg of xCe-yZn-Sr-MBGNs was immersed in 25 mL of pre-heated SBF solution at 37 °C with shaking at 120 rpm. After incubation, the xCe-yZn-Sr-MBGNs were collected by centrifugation, washed with ethanol and acetone to stop the reactions, and their morphology was analyzed using SEM (FE-SEM, Phenom Pharos Desktop FEG SEM, ThermoFisher Scientific, Waltham, MA, USA). FTIR was used to confirm apatite formation on the particle surface.

### 3.5. DPPH Radical Scavenging Assay

The antioxidant capacity of the xCe-yZn-Sr-MBGNs was evaluated using a DPPH radical scavenging assay. The DPPH (2,2-diphenyl-1-picrylhydrazyl, SRL, Mumbai, India) radical scavenging assay, a widely used method for assessing antioxidant capacity, was performed. Nanoparticle suspensions were prepared at concentrations of 1, 10, 25, and 50 mg/mL in methanol, and a 1 mM DPPH solution (OD ~1) was used. Ascorbic acid served as the positive control, while the DPPH solution without treatment was the negative control. Equal volumes (100 μL) of sample and DPPH solution were mixed in a 96-well plate and incubated in complete darkness at room temperature until observable changes occurred. Absorbance was recorded at 517 nm using a microplate reader (Infinite^®^ 200, Tecan Austria GmbH, Grödig, Austria). The percentage of inhibition was calculated using Equation (1):

(1) $$\mathrm{Percentage}\;\mathrm{of}\;\mathrm{inhibition}\;(\%)\;=\;\frac{A_{0}\;-\;A_{1}}{A_{0}}\;\times \;100$$

where A_0_ is the absorbance of the control, and A_1_ is the absorbance of the sample.

### 3.6. In Vitro Cytotoxicity

MC3T3-E1 cells, from a mouse pre-osteoblast cell line (ATCC^®^, CRL-2593 ™), were routinely cultured in α-MEM medium supplemented with 10% fetal bovine serum (FBS) and 100 U/mL Antibiotic-Antimycotic under standard conditions in a humidified atmosphere at 37 °C and 5% CO_2_. Cells were seeded into flat-bottomed 96-well plates (Corning^®^) at a density of 5 × 10^4^ cells/mL. Cells were incubated at 37 °C overnight to allow attachment and formation of a monolayer. The culture medium was then replaced with media containing nanoparticles at concentrations of 0–1 mg/mL (0, 10, 50, 100, 200, 300, 400, 500, 750, and 1000 μg/mL). Cells were exposed to the nanoparticles for 24 h and 72h (direct contact), while untreated cells served as the control (*n* = 6). Cell viability was assessed using the MTT colorimetric assay according to the manufacturer’s instructions, which was based on the reduction of 3-(4,5-dimethylthiazol-2-yl)-2,5-diphenyltetrazolium bromide to formazan. The soluble formazan in dimethyl sulfoxide (DMSO) was quantified using a microplate reader (Infinite^®^ 200, Tecan, Austria GmbH, Grödig, Austria) at 570 nm. Relative cell viability (% of the control) was expressed as the mean ± standard error of the mean.

### 3.7. In Vitro Mineralization

MC3T3-E1 cells were initially seeded into 24-well flat-bottom plates (Costar™, Corning™, New York, NY, USA) at a density of 5 × 10^4^ cells/mL. Upon attachment, cells were exposed to a treatment medium containing nanoparticles at 200 μg/mL: a concentration selected based on the 72 h MTT assay cut-off. This consistent seeding density, coupled with the non-toxic dosage, ensured uniform cell populations across groups for a direct comparison of mineralization potential driven by ionic stimulation. A basal and osteogenic medium (100 μg/mL L-ascorbic acid, 10 mM β-glycerophosphate, and 10 nM dexamethasone) served as negative and positive controls, respectively. Media were refreshed twice weekly. At days 7, 14, and 21, cells were fixed with 4% paraformaldehyde for 30 min at room temperature and stained with 2% (*w*/*v*) Alizarin Red S for 30 min, followed by washing with DI water. Calcium depositions appeared as red-stained regions. Mineralized nodules were visualized using an inverted optical microscope (LABOMED TCM400, Los Angeles, CA, USA) with the ToupView program. For semi-quantification, the bound dye was dissolved in DMSO at 37 °C, and absorbance was recorded at 430 nm using a microplate reader.

### 3.8. Osteogenic Differentiation

MC3T3-E1 cells were seeded in 6-well flat-bottom plates (Costar™, Corning™, NY, USA) at a density of 5 × 10^4^ cells/mL and exposed to medium-containing nanoparticles at 200 μg/mL. Cells cultured in basal complete media without nanoparticles and in media supplemented with osteogenic factors served as negative and positive controls, respectively. The culture medium was refreshed twice weekly over a 3-week period. At designated time points (7, 14, and 21 days), total RNA was extracted using the Monarch^®^ Total RNA Miniprep Kit (New England Biolabs, Ipswich, MA, USA) according to the manufacturer’s instructions. RNA concentrations were quantified using a NanoDrop 2000c Spectrophotometer (Thermo Fisher Scientific, Waltham, MA, USA). Complementary DNA (cDNA) was synthesized using the iScript™ cDNA Synthesis Kit (Bio-Rad Laboratories, Hercules, CA, USA) following the manufacturer’s protocol. Quantitative PCR (qPCR) analysis was then performed using the CFX96™ Real-Time PCR Detection System (Bio-Rad Laboratories, Hercules, CA, USA) with iTaq Universal SYBR Green Supermix (Bio-Rad Laboratories, Hercules, CA, USA). Primer sequences used in the experiment are listed in Table 4**.** Relative gene expression was calculated using the comparative 2^−ΔΔCt^ method, normalized to the reference gene *GAPDH*. All reactions were performed in triplicate.

### 3.9. Cell Migration

MC3T3-E1 cells were seeded in 6-well flat-bottom plates (Costar™, Corning™, NY, USA) at a density of 5 × 10^4^ cells/mL and standard cultured at 37 °C overnight to allow attachment. Cells were then treated with medium-containing nanoparticles at 200 μg/mL and incubated at 37 °C and 5% CO_2_ for 48 h to evaluate the sustained biological effect and cellular priming induced by the particles without the interference of particle-induced physical barriers during migration. A sterile pipette tip was used to make a straight scratch across the center of the cell monolayer, removing cells and creating a cell-depleted region. The plate was gently washed with PBS to remove any detached cells and cell debris and cultured in fresh completed α-MEM medium without particles. The plate was placed in a CO_2_ incubator, and images of the wound were captured at time intervals (0, 24, and 48h) using an inverted optical microscope (LABOMED TCM400, Los Angeles, CA, USA) with the ToupView program.

### 3.10. Antibacterial Testing

The antibacterial potential of the synthesized xCe-yZn-Sr-MBGNs was evaluated using the disk diffusion method against Gram-positive *Staphylococcus aureus* (*S. aureus* obtained from the Department of Microbiology, Faculty of Science, King Mongkut’s University of Technology Thonburi, Thailand) and Gram-negative *Pseudomonas aeruginosa* (*P. aeruginosa* obtained from the National Center for Genetic Engineering and Biotechnology, Pathum Thani, Thailand). Prior to testing, *S. aureus* and *P. aeruginosa* were grown overnight at 37 °C in Mueller–Hinton Broth (MHB), a standardized liquid growth medium optimized for antimicrobial susceptibility testing due to its specific mineral content and batch-to-batch consistency. Bacterial suspension was diluted with sterile saline to achieve a turbidity equivalent to 0.5 McFarland standards. The inoculum suspensions were streaked onto the Mueller–Hinton Agar (MHA) plate. The particle concentrations at 10 mg/mL were prepared in PBS under sterile conditions. Then, 20 μL of each sample was added to the sterile 6 mm diameter antibiotic assay disk (Whatman^®^, Cytiva, MA, USA). Penicillin–Streptomycin at 100 U/mL and PBS were used as the positive control and negative control, respectively. The inhibition zone was measured after incubation for 16–18 h.

### 3.11. Statistical Analysis

The size, calcium deposition, level of gene expression, antibacterial activity, and DPPH radical scavenging assay were analyzed using ANOVA and Tukey’s post hoc comparison test from R version 4.5.2 (R Core Team, Vienna, Austria) in RStudio version 2025.09.1 (Posit Software, PBC, Boston, MA, USA). The *p*-value < 0.05 was confirmed as statistically significant. Each experiment was conducted in triplicate, and the results are presented as the mean ± standard deviation (SD).

## 4. Conclusions

Spherical, monodisperse xCe-yZn-Sr-MBGNs (~200 nm) were successfully synthesized via a microemulsion-assisted sol-gel method with post-functionalization, exhibiting antibacterial, antioxidant, and osteogenic functionalities. Incorporation of Ce during synthesis promoted nanoceria formation within the mesoporous structure. All samples demonstrated bioactivity through apatite-like layer formation after immersion in SBF. Notably, the 1Ce-1Zn-Sr-MBGNs group yielded the most favorable outcomes overall, providing an optimal balance between therapeutic ion release and cellular viability. Co-doping with Ce and Zn produced multifunctional effects, enhancing calcium deposition, stimulating osteogenesis through upregulation of osteogenic gene expression, and improving cell migration. While higher concentrations (2Ce-1Zn-Sr-MBGNs) showed the strongest antibacterial inhibition against *P. aeruginosa* and *S. aureus* and the highest DPPH radical scavenging capacity, the 1Ce-1Zn-Sr-MBGNs formulation was identified as the most effective for bone regeneration due to its superior osteogenic induction without compromising cell population density. This study establishes the multifunctionality of xCe-yZn-Sr-MBGNs through a series of in vitro assessments. While the current findings provide a robust foundation, further investigation is needed to fully validate their performance for clinical applications. This includes performing clinically relevant antibacterial evaluations, such as MIC/MBC and biofilm adhesion assays, as well as quantifying intracellular ROS levels to fully elucidate the Ce^3+^/Ce^4+^ redox interactions within the complex cellular microenvironment. Transitioning to in vivo bone regeneration models will be a critical next step in validating the therapeutic efficacy of these nanoparticles. Such advancements will be pivotal in bridging the gap between experimental development and the clinical translation of xCe-yZn-Sr-MBGNs for regenerative medicine. 

## Figures and Tables

**Table 1:** Hydrodynamic size and particle size determined by SEM and Zeta potential for the synthesized xCe-yZn-Sr-MBGNs.

Samples	Hydrodynamic Size (nm)	Size SEM (nm)	Zeta Potential (mV)
0Ce-0Zn-Sr-MBGNs	277.6 ± 10.8	189.6 ± 12.7	−27.5 ± 1.0
1Ce-0Zn-Sr-MBGNs	285.4 ± 9.5	186.1 ± 10.9	−21.3 ± 1.0
1Ce-0.5Zn-Sr-MBGNs	261.8 ± 12.3	189.4 ± 11.8	−14.1 ± 1.5
1Ce-1Zn- Sr-MBGNs	243.9 ± 11.7	187.9 ± 11.7	−13.1 ± 0.7
2Ce-0Zn-Sr-MBGNs	272.8 ± 10.2	170.9 ± 12.7	−18.8 ± 0.8
2Ce-0.5Zn-Sr-MBGNs	259.1 ± 13.1	168.0 ± 19.9	−18.2 ± 0.6
2Ce-1Zn-Sr-MBGNs	282.2 ± 13.9	172.7 ± 16.3	−16.7 ± 0.3

**Table 2:** The textural properties of xCe-yZn-Sr-MBGNs.

Samples	BET Surface Area (m^2^/g)	Average Pore Diameter (nm)	Pore Volume (cm^3^/g)
0Ce-0Zn-Sr-MBGNs	372.5	11.6	17.2
1Ce-0Zn-Sr-MBGNs	423.4	9.2	16.1
1Ce-0.5Zn-Sr-MBGNs	385.3	9.6	17.0
1Ce-1Zn- Sr-MBGNs	340.1	10.2	16.2
2Ce-0Zn-Sr-MBGNs	377.2	12.0	16.9
2Ce-0.5Zn-Sr-MBGNs	360.4	11.9	17.3
2Ce-1Zn-Sr-MBGNs	381.3	11.2	16.9

**Table 3:** The normative ratio of xCe-yZn-Sr-MBGNs (mol%).

Samples	The Nominal Ratio (mol%)				
	SiO_2_	CaO	SrO	Ce_2_O_3_	ZnO
0Ce-0Zn-Sr-MBGNs	60	20	20	-	-
1Ce-0Zn-Sr-MBGNs	60	20	19	1	-
1Ce-0.5Zn-Sr-MBGNs	60	20	18.5	1	0.5
1Ce-1Zn-Sr-MBGNs	60	20	18	1	1
2Ce-0Zn-Sr-MBGNs	60	20	18	2	-
2Ce-0.5Zn-Sr-MBGNs	60	20	17.5	2	0.5
2Ce-1Zn-Sr-MBGNs	60	20	17	2	1

**Table 4:** Primers sequences used for real-time quantitative PCR (qPCR) analysis.

Gene	Primer Sequences
*RUNX-2*	F: 5′ TTACTTACACCCCGCCAGTC 3′ R: 5′ CACTCTGGCTTTGGGAAGAG 3′
*ALP*	F: 5′ CGGGACTGGTACTCGGATAA 3′ R: 5′ TGAGATCCAGGCCATCTAGC 3′
*COL1A1*	F: 5′ GAGCGGAGAGTACTGGATCG 3′ R: 5′ GTTCGGGCTGATGTACCAGT 3′
*OCN*	F: 5′ TGACCTCACAGATGCCAAGC 3′ R: 5′ CGCCGGAGTCTGTTCACTAC 3′
*OPN*	F: 5′ GCCGAGGTGATAGTGTGGTT 3′ R: 5′ TGAGGTGATGTCCTCGTCTG 3′
*GAPDH*	F: 5′ CTACCCCCAATGTGTCCGTC 3′ R: 5′ GGCCTCTCTTGCTCAGTGTC 3′

## Data Availability

The data that supports the findings of this study are available from the corresponding author upon reasonable request.
